# Comparative Analysis of Impulsivity Profiles in Children and Young Patients with Anorexia Nervosa Restrictive Type, Anorexia Nervosa Binge Eating/Purging Type, Bulimia Nervosa, and Binge Eating Disorder

**DOI:** 10.1192/j.eurpsy.2024.261

**Published:** 2024-08-27

**Authors:** M. D. M. Arango, E. Montasell

**Affiliations:** ^1^Psychiatry; ^2^Neuropsychology, ITA Clinic, Barcelona, Spain

## Abstract

**Introduction:**

High levels of impulsivity are associated with individuals suffering from eating disorders. Impulsivity is a complex and multidimensional construct, with elevated impulsivity traits posing a specific risk in relation to binge-eating and purging disorders when compared to restrictive types eating disorders.

**Objectives:**

Our aim was to identify the difference in impulsivity profile in children and young patients with eating disorders, including anorexia nervosa restrictive type (ANR), anorexia nervosa binge eating/purging type (ANP), bulimia nervosa (BN) and binge-eating disorder (BED).

**Methods:**

Patients aged 21 years or younger, meeting the DSM-V criteria for ANR (n=125), ANP (n=48), BN (n=38), and BED (n=37) were enrolled in the study. The participants had an average age of 16.3 ± 2.15 years. Data collection involved the administration of the UPPS Impulsiveness Scale (UPPS). Bilateral Student’s t-tests were conducted to evaluate potential statistically significant differences among the diagnostic groups.

**Results:**

Our results indicated statistically significant differences in total impulsivity between patients diagnosed with ANR and each of the other eating disorders including ANP (T-Stud -2,19 *p* < .02), BN (T-Stud -2.17*p* < .03), and BED (T-Stud -2,68 *p* < .008) (Figure 1). However, no significant differences were observed among the other eating disorder groups. Nevertheless, it is noteworthy that heightened impulsivity traits, particularly sensation-seeking tendencies, were a common feature among all subtypes of eating disorders, regardless of their specific diagnostic category. Impulsivity and age also exhibited a statistically significant negative correlation (r = -0.13, *p* = .03) (Figure 2).

**Image:**

**Figure:**
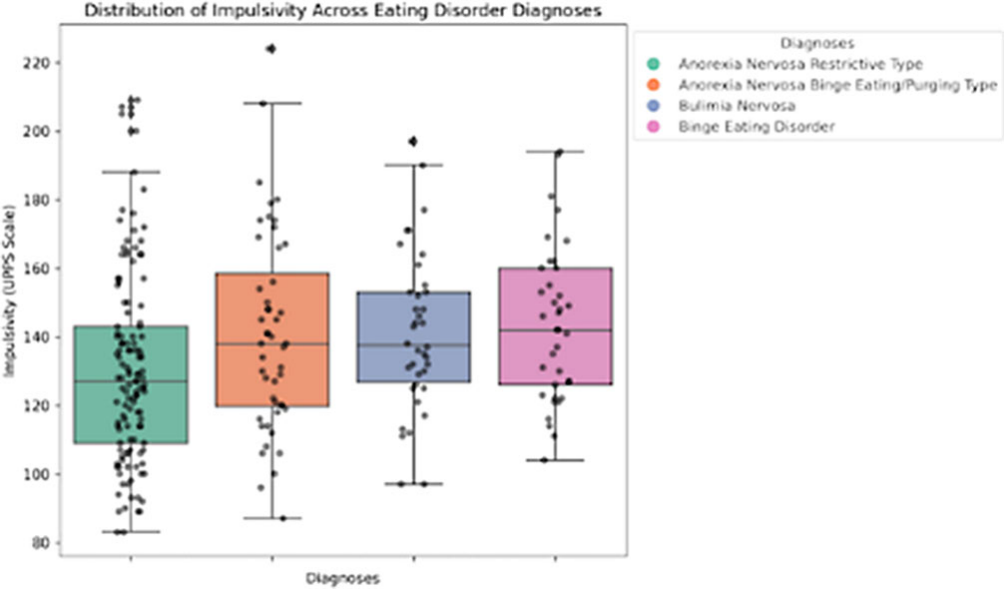

**Image 2:**

**Figure:**
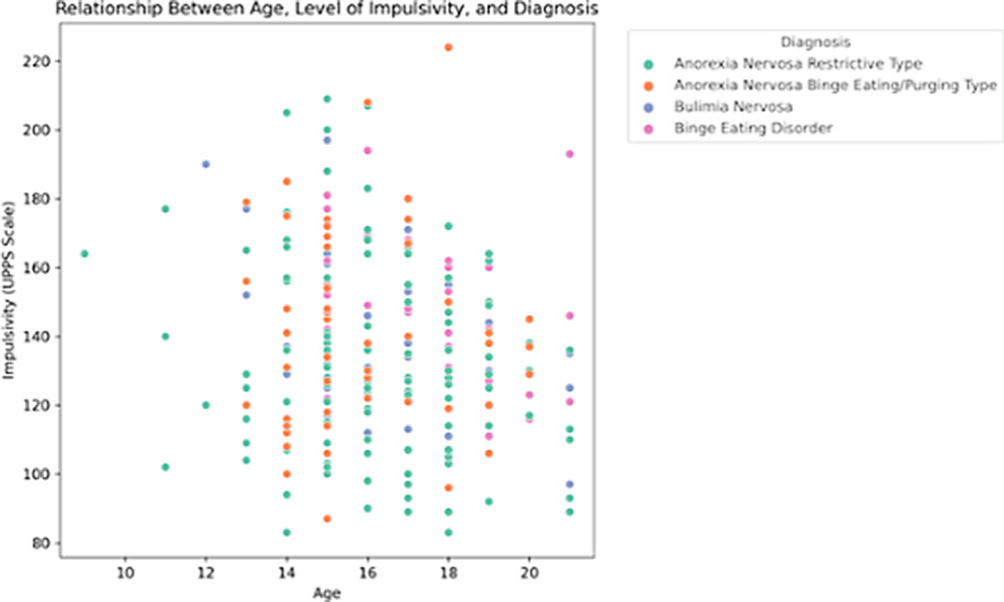

**Conclusions:**

Impulsivity in individuals with restrictive and binge/purgative eating disorders differ significantly, with lower levels of impulsivity in ANR (Figure 1), except for sensation-seeking tendencies. This suggests that both groups may share a similar inclination for seeking intense emotions or engaging in emotionally arousing behaviors. As individuals age from adolescence to young adulthood, there is a tendency for impulsivity levels to decrease (Figure 2).

**Disclosure of Interest:**

None Declared

